# Integrative Analysis of Porcine microRNAome during Skeletal Muscle Development

**DOI:** 10.1371/journal.pone.0072418

**Published:** 2013-09-11

**Authors:** Lijun Qin, Yaosheng Chen, Xiaohong Liu, Sanxing Ye, Kaifan Yu, Zheng Huang, Jingwei Yu, Xingyu Zhou, Hu Chen, Delin Mo

**Affiliations:** State Key Laboratory of Biocontrol, School of Life Sciences, Sun Yat-Sen University, Guangzhou, P. R. China; University of Minnesota Medical School, United States of America

## Abstract

Pig is an important agricultural animal for meat production and provides a valuable model for many human diseases. Functional studies have demonstrated that microRNAs (miRNAs) play critical roles in almost all aspects of skeletal muscle development and disease pathogenesis. To investigate the miRNAs involved in regulating different periods of skeletal muscle development, we herein performed a comprehensive research for porcine microRNAome (miRNAome) during 10 skeletal muscle developmental stages including 35, 49, 63, 77, 91 dpc (days post coitum) and 2, 28, 90, 120, 180 dpn (days postnatal) using Solexa sequencing technology. Our results extend the repertoire of pig miRNAome to 247 known miRNAs processed from 210 pre-miRNAs and 297 candidate novel miRNAs through comparison with known miRNAs in the miRBase. Expression analysis of the 15 most abundant miRNAs in every library indicated that functional miRNAome may be smaller and tend to be highly expressed. A series of muscle-related miRNAs summarized in our study present different patterns between myofibers formation phase and muscle maturation phase, providing valuable reference for investigation of functional miRNAs during skeletal muscle development. Analysis of temporal profiles of miRNA expression identifies 18 novel candidate myogenic miRNAs in pig, which might provide new insight into regulation mechanism mediated by miRNAs underlying muscle development.

## Introduction

Pork is an important source of protein for human consumption, particularly in China where it tops the list of meat consumed. In addition, the pig can be considered as a suitable model for human diseases because it is similar with human in size, physiology, pathology and genomics [1]. Muscle development is a complex process, including not only embryonic myogenesis but adult myofiber maturation. Most research suggested that porcine primary myofibers mainly formed at 35 to 64 dpc and secondary myofibers at 54 to 90 dpc [2]. However, Swatland et al. pointed out that fetal myofiber hyperplasia ceased at slightly later at 70 days gestation and the increase in number of myofibers seen in a transverse section was caused by growth in length of myofibers [3]. Similarly, Zhao et al. discovered that myogenesis was almost completed before 77 dpc [4], suggesting that the muscle ﬁber formed at 35 to 77 dpc. Other research described that there existed a third generation of fibers around birth in pigs and might be part of mechanisms leading to the larger muscle mass [5,6]. The total fiber number (TFN) does not increase after birth and is fixed at the number formed during embryonic life. Myofibers were classified according to their contractile and metabolic properties as type 1, type 2 (2A and 2B) and an intermediate type 2X. From late gestation through the first four postnatal weeks, myofibers underwent a process of maturation from type 1 to type 2 [4,6]. Hypertrophy is another process of skeletal muscle maturation, involving an increase in muscle mass and in the size, associated with increased myofibrillar protein content. Signaling pathways such as insulin-like growth factor I (IGF-I) and Ca^2+^/calmodulin-dependent transcriptional pathways have been demonstrated to govern skeletal muscle hypertrophy and atrophy [7]. Understanding the complex mechanism underlying porcine muscle development might contribute to swine industry as well as human muscular growth and diseases.

Increasing evidence suggests that miRNAs serve as biological regulators by mediating gene expression. It was increasingly clear that miRNAs involve almost all aspects of skeletal muscle development, including cell migration, proliferation, differentiation and apoptosis [8,9]. Despite the significant role of miRNAs in the regulation of myogenesis, the miRBase (release 18.0) lists only 257 distinct mature miRNA sequences in pigs, significantly fewer than that in human. It’s worth noting that many miRNAs are expressed in a tissue-specific or stage-specific manner [10], and the best-characterized muscle-specific miRNAs (myomiRs [11]) are miR-1, miR-206 and miR-133 families which specifically expressed in cardiac and skeletal muscles. Dicer was known to be essential for miRNA biogenesis, whose elimination in the skeletal muscles could lead to prenatal death, muscle mass reduction or myofiber malformation [12], an outcome highlights the significance of miRNAs in muscle development. Currently, scientists tend to study the molecular mechanism of swine skeletal muscle development through analysis of miRNA transcriptome profiles [13,14], discovering and identifying more miRNAs based on bioinformatics analysis. However, it is still not enough for investigation in mapping a nearly complete porcine miRNAome during skeletal muscle development.

The Landrace, one of the most popular pig breeds in most countries in Europe, North America and Asia, was employed in this investigation. Its miRNAome during skeletal muscle development at ten developmental stages including five prenatal stages (35, 49, 63, 77, 91 dpc, simplified as LR 1, 2, 3, 4, 5) and five postnatal stages (2, 28, 90, 120, 180 dpn, simplified as LR 6, 7, 8, 9, 10) using Solexa sequencing. The time points cover almost all morphological and physiological changes of porcine skeletal muscle growth and development [4]. Thus, this study will provide a thorough investigation of miRNAome in porcine skeletal muscle to facilitate a better understanding of their involvement in myogenesis.

## Materials and Methods

### Ethics statement

All animal procedures were performed according to guidelines developed by the China Council on Animal Care and protocols were approved by the Animal Care and Use Committee of Guangdong Province, China. The approval ID or permit numbers are *SCXK* (*Guangdong*) *2004-0011* and *SYXK* (*Guangdong*) *2007-0081*.

### Sample preparation

Fifteen Landrace (LR) purebred sows with the same genetic background were artificially inseminated with semen from the same purebred boars. The pregnant sows were slaughtered at five prenatal stages (35, 49, 63, 77, 91 dpc, days post coitum) while the piglets and adult pigs were slaughtered at five postnatal stages (2, 28, 90, 120, 180 dpn, days postnatal). The longissimus dorsi muscle tissues collected per time point were used as the experimental samples for sequencing. These samples were snap-frozen in liquid nitrogen and stored at -80°C.

### Small RNA library construction and Solexa sequencing

Total RNA was extracted using miRNeasy Mini Kit (Cat#217004, QIAGEN, GmBH, Germany) according to the manufacturer’s protocol. For each developmental stage, equal quantities of total RNA isolated from three individual pigs were pooled. Total RNA integrity was measured on an Agilent 2100 Bioanalyzer system (Agilent) for quality control.16-35 nt RNA fragments were excised, purified from a PAGE gel, and ligated with 5′ and 3′ adaptors using T4 RNA ligase. Reverse transcription followed by PCR was used to create cDNA constructs based on the small RNA ligated with 3′ and 5′ adapters. Subsequently, the amplified cDNA constructs were purified from agarose gel, in preparation for sequencing analysis using the Illumina Genome Analyzer (Illumina, CA, USA) according to the manufacturer’s instructions.

### Data analysis

The raw data were processed using Illumina 1G Genome Analyzer Pipeline software and then submitted to data filtration. Clean reads were obtained after filtering low-quality reads and trimming the adaptor sequences. All of the clean reads were initially searched against miRBase (version 18; http://www.mirbase.org/) to identify known porcine miRNAs. Un-mappable reads subsequently were annotated and classified by reference to non-coding RNAs in the Ensemble (ftp://ftp.ensembl.org/pub/release-69/fasta/sus_scrofa/ncrna/), piRNA (http://pirnabank.ibab.ac.in/) and Rfam (version 10; http://rfam.sanger.ac.uk/) databases. The mappable sequences were achieved and used for further analysis. Meantime, many unannotated sequences that cannot match any above databases were analyzed by miRDeep [15] (http://deepbase.sysu.edu.cn/miRDeep.php) to predict novel miRNA candidates. Their hairpin structures were then analyzed using RNAfold software (http://rna.tbi.univie.ac.at/cgi-bin/RNAfold.cgi). Only with tipical stem-loop hairpins and the free energy lower than -20 kcal/mol could the sequences be considered to be potential novel miRNAs. After all annotation steps, the sequencing libraries were used for size distribution and saturation analysis. All the sequence data have been submitted to the NCBI Sequence Read Archive (http://www.ncbi.nlm.nih.gov/Traces/sra/) under accession No.SRA073195.

### STEM Clustering

STEM (Short Time-series Expression Miner v 1.1) herein was used for clustering and visualizing possible profiles of differentially expressed (DE) miRNAs’ change in expression over time (less than 8 time points). The Maximum Unit Change in Model Profiles between Time Points was adjusted to 1 and the Maximum Number of Model Profiles to 50. MiRNA expression profiles were clustered according to the correlation coefficient. The statistical signiﬁcance of the number of genes assigned to each proﬁle versus the number expected was computed by algorithm suggested by Ernstet et al. [16] and the default P-value was 1E-5. Statistically signiﬁcant model proﬁles which are similar to each other can be grouped together to form clusters of proﬁles.

### Target Prediction and KEGG Orthology Analysis

Based on the sequences of the miRNAs, the targets of DE miRNAs were predicted using the online database miRecords [17] (http://mirecords.biolead.org/) and chosen when they were predicted in at least databases integrated in miRecords. The predicted target genes were subsequently submitted to KOBAS [18] for KEGG Orthology analysis (http://kobas.cbi.pku.edu.cn/home.do) using KEGG database. Given the P values in KOBAS, pathways with statistically significant values (P<0.05) were chosen. In addition, SPSS was used to divide the pathways into categories by using the fuzzy theories to K-means algorithm.

### Stem-loop RT-PCR

To validate the sequencing results, nine miRNAs with different expression levels were selected and Stem–loop RT-PCR was performed using the Lightcycler480 (Roche) with SYBR-Green detection (SYBR PrimeScript RT-PCR Kit, TaKaRa Biotechnology Co. Ltd.) according to the manufacturer’s instructions. The reverse transcriptase reaction was performed using Reverse Transcription System Kit (Promega) with the stem-loop primers listed in Table S7. Each real-time PCR system contained 5 µl 2×SYBR Premix DimerEraser^TM^ (TaKaRa), 0.3 µl forward and reverse primers respectively, 1 µl template cDNA, and dH_2_O up to the final volume of 10 µl. The reactions were incubated at 95°C for 30 s, followed by 40 cycles of 95°C for 10 s, 60°C for 20 s and 72°C for 10 s. Mir-17-5p was chosen as internal control and all reactions were run in triplicate. The experimental data were analyzed using the 2^-∆∆CT^ method.

## Results

### Overview of sequencing data

After trimming of adaptor sequences and removal of low quality reads, 181,224,007 total clean reads were obtained from ten libraries, of which almost 96% matched small RNA databases mentioned in the Materials and Methods, namely annotated reads (Table S1). Saturation plots of ten libraries were created to assess the efficiency and accuracy of deep sequencing for miRNA detection (Figure S1). Taking the LR1 library as an example, the deeper were sequenced, the more unique small RNAs were found. However, the most of unique small RNAs were unknown, for the number of annotated small RNAs accounted for less than 20%. Furthermore, the number of new sequences observed for known small RNAs and porcine miRNAs (miRBase found) reached a plateau when the number of sequenced reads was 9,500,000, suggesting that the library capacity approached saturation. Similar plots of the other nine libraries were shown in Figure S1. Therefore, the results demonstrated that the deep sequencing data were able to accurately represent the miRNA transcriptome profiles of porcine skeletal muscle.

**Figure 1:**
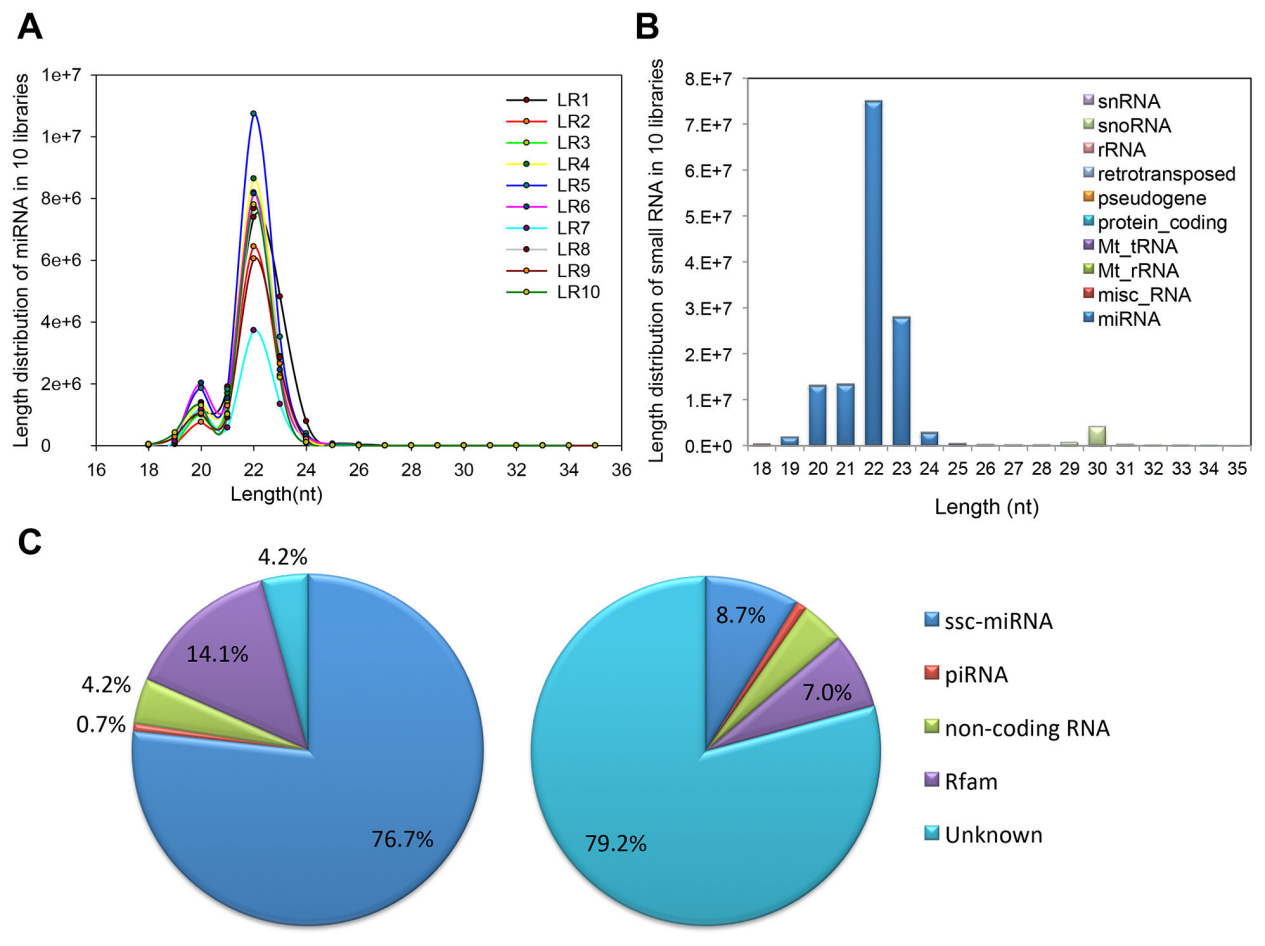
Basic analysis of Sequencing data. **A**–**B**. Sequence length distribution of known miRNAs (A) and small RNAs (B); C. Count number distribution of Total Reads (left) and Unique Small RANs (right).

Of the annotated sequences, the most abundant size class in the small RNA sequences distribution was 22 nt, followed by 21 and 23nt (Figure 1A and B), consistent with the known 21-23 nt range for mature miRNAs. In addition, the annotated sequences were analyzed referencing the data from miRBase (release 18.0, containing 228 precursors and 257 mature miRNAs), resulting 247 (96%) mature miRNAs and 210 (92%) precursor were identified. Next, the rest annotated sequences were subjected to piwi-interacting RNA (piRNA) database, Rfam (version 10) and non-coding RNA in Ensemble and the results were summarized in Table S1. Most annotated small RNAs were porcine miRNAs (ssc-miRNAs), accounting for 76.7% of the total sequence reads but a less proportion (8.7%) of the unique sequence reads in ten small RNA libraries (Figure 1C). The results indicated that the majority of small RNAs were annotated miRNAs while other classes added diversity. Together, all of the above results provide confidence that the deep sequencing data were highly enriched for known miRNA sequences, suggesting that the data are reliable for analyzing miRNA expression profiles as well as for predicting novel miRNAs.

### Identification of potential novel miRNAs

Deep sequencing is a robust approach for discovering novel miRNAs that are expressed at low levels. To find more potential miRNAs, unannotated sequences longer than 18 nt were searched against porcine genome and analyzed by miDeep. According to criteria for novel miRNAs identification, 297 candidate novel miRNAs were predicted and named ssc-miR-new-N (N=1 ~ 297). These novel miRNAs ranged from 20 to 23 nt in length, among which 22 nt sequences accounted for the most (40%) followed by23 nt (25%), 21 nt (22%) and 20nt (13%), consistent with typical length distribution of mature miRNAs. Total reads of these novel miRNAs counted up from ten libraries were at a relatively low level: 267 putative novel miRNAs were sequenced below 100 times, 27 were below 1000 times (of which 3 were above 500 times), 2 were above 1000 times and only 1 were above 10000 times (Table S2). Hairpin structures of the putative novel miRNAs were then analyzed using RNAfold.

### Characterization of miRNAome in different stages of skeletal muscle

As presented in Table S3, 247 identified mature porcine miRNAs have been substituted as 257 mature porcine miRNAs (If the normalized expression of a given miRNA is zero, its expression value will be modified to 0.01) that expressed at different levels, ranging from single reads for the least abundant to millions of reads for the most abundant. Previous study has demonstrated that over 60% of miRNAs detected by deep sequencing had no discernible activity, all of which expressed below 100-1,000 reads per million (RPM) [19]. Thus, the distribution of numbers for normalized miRNAs was summarized in Table S3, from which we could find that the number of miRNAs with mean expression value below 1000 is 197 (123 and 74, respectively), almost accounts for 77% of the total number of miRNAs (48% and 29%, respectively) in the ten libraries. However, the number of miRNAs expressed on average over 1000 RPM is 60 (23%), among which 15 miRNAs (6%) expressed over 10000 RPM. Moreover, we counted the top most abundant 15 miRNAs in each library, accounting for 85% of the total counts of all unique miRNAs on average (Figure S2), from which we obtained a common 27 miRNAs with the highest expression level in ten libraries (Table 1, details see Table S4), suggesting that the majority of abundant miRNAs are from fewer miRNAs [20]. Previous studies have announced that there were more miRNAs with reported myogenic function in muscle besides well-known myomiRs (Table 2). Among the 27 miRNAs, we found 11 (40%) miRNAs have a relationship with muscle development validated by experiments. Almost all known myomiRs were identified with the most abundance. The most abundant miRNA was ssc-miR-1, which presented by more than 2,100,000 RPM in ten libraries. The predominance of miR-1 is consistent with its well established function during skeletal muscle development [21] and reported role during porcine myogenesis [22]. Similarly, two other myomiRs, miR-133 [21] and miR-206 [23], were highly expressed and ranked the 4^th^ and 6^th^ respectively, while two other miRNAs (miR-378 [24,25] and miR-143 [25]) ranked the 2^nd^ and 3^rd^ have been identified to participate in the proliferation and differentiation of muscle cells.

**Table 1:** The top most abundant 15 miRNAs in each library.

**Rank**	**Mature miRNA**	**Total normalizedexpression (RPM**)	**35 dpc (LR1**)	**49 dpc (LR2**)	**63 dpc (LR3**)	**77 dpc (LR4**)	**91 dpc (LR5**)	**2 dpn (LR6**)	**28 dpn (LR7**)	**90 dpn (LR8**)	**120 dpn (LR9**)	**180 dpn (LR10**)
1	ssc-miR-1	2103431.74	14168.38	79554.80	117686.25	126702.52	119640.12	142618.55	218084.74	310634.60	402667.63	571674.13
2	ssc-miR-378	1625963.19	19043.59	104583.43	93292.67	101497.98	121417.03	283735.95	329605.58	377885.10	79332.57	115569.27
3	ssc-miR-143-3p	1176846.35	85966.88	97786.87	101871.98	126658.26	132892.15	150534.02	172399.50	96310.23	112916.20	99510.27
4	ssc-miR-133a-3p	607495.75	2727.83	15804.31	26969.46	18529.50	53763.56	92575.68	73767.14	80230.65	160502.23	82625.41
5	ssc-miR-30a-5p	531375.43	96303.29	82081.00	114727.74	91093.80	66594.81	29197.53	22315.70	10041.35	12723.78	6296.41
6	ssc-miR-206	486805.67	14430.69	58818.28	70883.78	80508.60	114389.28	53764.91	31347.29	18392.07	28534.43	15736.34
7	ssc-let-7f	317168.48	14823.77	51717.88	51980.72	53943.49	51279.82	19763.81	17837.04	12813.37	25372.54	17636.05
8	ssc-miR-148a	253477.02	48633.91	62835.68	30149.73	58289.18	25638.64	12373.55	5875.23	4296.70	3018.60	2365.82
9	ssc-miR-10b	234732.07	55274.96	42326.43	30113.66	23607.22	21081.96	14529.22	14327.48	10202.41	13349.09	9919.64
10	ssc-miR-127	214267.02	11872.22	62283.84	56300.94	44626.90	20717.40	10194.69	4062.24	1229.49	2479.72	499.59
11	ssc-miR-140*	187553.50	163247.38	4841.03	4036.55	4056.84	2828.27	1901.68	1341.56	893.49	2227.68	2179.02
12	ssc-miR-30d	172056.17	31767.19	29605.00	30624.26	20723.44	20029.29	15681.53	10186.79	4142.37	6753.67	2542.63
13	ssc-miR-542-3p	155142.75	20257.32	32769.39	36970.07	30137.30	18650.10	6800.57	4061.40	1364.72	3414.29	717.59
14	ssc-miR-21	131742.03	32846.54	22577.72	16102.99	19634.04	12044.42	5700.21	5369.31	7447.14	6139.29	3880.37
15	ssc-let-7a	121288.75	12151.08	18169.62	17302.43	20377.25	19469.48	8340.53	7167.23	4962.23	8175.71	5173.18
16	ssc-miR-30e-5p	87618.91	20876.82	14889.87	15178.98	11951.46	7061.26	5816.45	3735.71	1871.88	4270.27	1966.20
17	ssc-miR-101	75739.16	10927.72	10465.49	11372.56	10378.67	8792.07	9002.56	4594.39	2650.42	4438.02	3117.25
18	ssc-miR-10a	73489.75	54439.05	6034.88	3408.41	2594.58	1945.94	1289.80	1131.32	476.95	1178.30	990.51
19	ssc-miR-26a	70050.82	4336.72	5112.43	5343.07	6004.85	8431.37	9595.40	6413.11	5718.05	11470.45	7625.36
20	ssc-miR-103	64540.89	24347.45	11077.11	6324.15	6941.08	6807.53	3326.21	1585.72	1207.70	2095.07	828.87
21	ssc-miR-30c	58032.92	6600.70	4842.93	5957.25	4372.65	6574.03	8852.00	4789.69	4229.47	8277.57	3536.63
22	ssc-miR-133b	57966.15	1095.91	3365.47	4991.24	4548.17	11303.05	11654.61	3570.98	3863.09	9805.15	3768.48
23	ssc-miR-411	35786.91	625.66	3329.22	5246.32	5586.72	11947.48	4728.62	369.80	137.92	3719.29	95.87
24	ssc-miR-30e-3p	35683.28	5911.43	5695.56	3643.81	2647.77	2052.03	2131.20	4285.46	3967.09	1926.72	3422.21
25	ssc-miR-9-2	28464.42	27754.31	103.78	63.66	31.91	27.52	12.07	5.86	17.92	178.08	269.31
26	ssc-miR-9-1	28464.22	27754.31	103.40	63.33	32.18	27.79	11.86	5.86	18.09	178.67	268.70
27	ssc-miR-30b-5p	28196.90	1534.35	1614.82	2019.87	1761.57	2834.33	5252.29	3199.22	2536.32	5126.60	2317.53

**Table 2:** Information of miRNAs known to function in muscle development (muscle-related miRNAs).

**Transcription Factors (TF**)** & Signaling pathways**	**microRNA (miR**)	**TF - miR Relationship**	**Targets (including Potential Targets**)	**MiR - targets Relationship**	**Tissues &Cells**
MyoD [62–64], MEF2 [62,65–67], SRF [62], Myogenin [63], Myf5 [63], MRF4 [63], Twist [65], mTOR [68], SREBP [66]	miR-1	up-regulation	HDAC4 [21], Cx43 [69,70], G6PD [71], Sox6 [72], Hand2 [62], KCNJ2 [69], HSP60 [73], HSP70 [73], caspase-9 [73], c-Met [74], Pax7 [75], Pax3 [64], IGF-1R [76], KLF4 [77]	down-regulation	C2C12, skeletal muscle, cardiac muscle, smooth muscle, Rhabdomyosarcoma
HMOX1 [78], Notch3 [67]		down-regulation	Notch3 [67]	down-regulation	
Myogenin [63], MyoD [63], MEF2 [66], Myf5 [63], MRF4 [63], SREBP [66]	miR-133	up-regulation	SRF [21,79], KLF15 [79], nPTB [80], UCP2 [81], RhoA [82], Cdc42 [82], Nelf-A/WHSC2 [82], CTGF [83], HSP60 [73], HSP70 [73], caspase-9 [73], Runx2 [84], dynamin 2 [85]	down-regulation	C2C12, skeletal muscle, cardiac muscle, Rhabdomyosarcoma
HMOX1 [78]		down-regulation		down-regulation	
Myogenin [63], MyoD [63,64,86], Myf5 [63], MRF4 [63], MEF2C [67]	miR-206	up-regulation	Cx43 [70], HDAC4 [87], DNA polymerase [88], Fstl1 [86], Utrn [86], c-Met [74], Pax7 [89], Pax3 [64], Hmgb3 [90], Otx2 [91], TIMP3^#^ [92], VEGF^#^ [93]	down-regulation	C2C12, skeletal muscle, Rhabdomyosarcoma, laryngeal squamous cell carcinoma (LSCC)
TGF-β [87], BMP2 [94], HMOX1 [78], Notch3 [67]		down-regulation	Notch3 [67]	down-regulation	
MyoD [24]	miR-378	up-regulation	MyoR [24], BMP2^*^ [53], MAPK1^*^ [53]	down-regulation	C2C12, skeletal muscle^*^
IGF1 [95]		down-regulation	IGF1R [95]	down-regulation	cardiac muscle
	miR-155		MEF2A [96], OLFML3^*^ [97]	down-regulation	skeletal muscle^*^, vascular smooth-muscle
KDM2B (Ndy1/FBXL10/JHDM1B) [98]	let-7/miR-101	down-regulation	EZH2 [98]	down-regulation	skeletal muscle
	let-7b		GHR [99]	down-regulation	
STAT3 [100]	miR-124a	up-regulation			embryonic stem cell (ESC)
mTOR [34]	miR-125	down-regulation	IGF-II [34], Cbx7 [101], SP7 [102]	down-regulation	skeletal muscle, smooth muscle, ESC
	miR-126		Spred-1 [58], VCAM-1 [58], IRS-1 [57]	down-regulation	cardiac muscle, ESC
	miR-128a		PPARγ [36], Runx1 [36], Pax3 [36]	down-regulation	skeletal muscle
	miR-130a		GAX [103], HOXA5 [103]	down-regulation	vascular endothelial cells (ECs)
	miR-135		Smad5 [84], JAK2^#^ [104]	down-regulation	C2C12, Hodgkin lymphoma
SRF [105], myocardin [105]	miR-143/145	up-regulation	SRF [25], myocardin [25], Nkx2-5 [25]	down-regulation	smooth muscle
	miR-144		IRS1 [106]	down-regulation	Type II diabetes mellitus
	miR-148a		ROCK1 [27]	down-regulation	skeletal muscle
	miR-15a		DLK1 [107]	down-regulation	3T3-L1 preadipocytes
	miR-15a/16		cyclin D1 [108]	down-regulation	skeletal muscle
	miR-181		Hox-A11 [26], Cbx7 [101]	down-regulation	skeletal muscle, ESC
	miR-199a		Hif-1α [37], Sirt1 [37]	down-regulation	cardiac muscle
	miR-204		Runx2 [109]	down-regulation	smooth muscle
	miR-208a		Thrap1 [110], myostatin [110]	down-regulation	cardiac muscle
	miR-208b/499		Sox6 [11], Purβ [11], Sp3 [11], HP-1β [11]	down-regulation	muscle
TGF-β/BMPs signaling [111], p38 [112], MKP-1 [112]	miR-21	up-regulation	PTEN [113], PDCD4 [111]	down-regulation	skeletal muscle, cardiac muscle, smooth muscle
Hif1a [114]	miR-210	up-regulation			cardiac muscle
Myogenin [115], MyoD [115]	miR-214	up-regulation	Ezh2 [115], N-Ras [40]	down-regulation	C2C12, skeletal muscle, ESC
Ras-MAPK pathway [44]	miR-221	up-regulation	p27 [44], Mdm2 [116]	down-regulation	skeletal muscle, mesenchymal cells
Ras-MAPK pathway [44]	miR-222	up-regulation	p27 [44], β1-syntrophin [117]	down-regulation	skeletal muscle
NFATc3 [118]	miR-23a		MAFbx/atrogin-1 [33], MuRF1 [33,118], Myh1/2/4 [32]	down-regulation	skeletal muscle
TGF-β [35]	miR-24	down-regulation			skeletal muscle
C/EBPα [119]	miR-26a	up-regulation	GSK-3β [119], Ezh2 [29], Smad1 [28], Smad4 [28]	down-regulation	skeletal muscle, smooth muscle
Pitx2 [31]	miR-27a/b	down-regulation	MSTN [30], Pax3 [31], myostatin [30]	down-regulation	skeletal muscle
NF-kappa-B-YY1 [41,43], CKD [42], TGF-beta-Smad3 signaling [120]	miR-29	down-regulation	HDAC4 [87], YY1 [41,42], Smad3 [87], Smad4 [121], MMP-2 [43], COL1A1 [122], ELN [122], Rybp [123]	down-regulation	C2C12, skeletal muscle, cardiac muscle, Aneurysm
	miR-30		CTGF [83]	down-regulation	cardiac muscle
	miR-31		Myf5 [124]	down-regulation	skeletal muscle, Duchenne muscular dystrophy
	miR-320		PFKm [125]	down-regulation	muscle
	miR-322/424		Cdc25A [38]	down-regulation	skeletal muscle
	miR-351		E2f3 [126]	down-regulation	skeletal muscle
SRF [39], MRTF-A [39], MyoD [39]	miR-486	up-regulation	PTEN [39], Foxo1 [39], Pax7 [89]	down-regulation	skeletal muscle
	miR-489		Dek [127]	down-regulation	skeletal muscle
	miR-494		mtTFA [128], Foxj3 [128]	down-regulation	skeletal muscle
	miR-503		Cdc25A [38,129], CCNE1 [129]	down-regulation	skeletal muscle
	miR-546		Mybbp1a [130]	down-regulation	skeletal muscle
	miR-669a/669q		MyoD [131]	down-regulation	skeletal muscle
	miR-696		PGC-1α [132]	down-regulation	skeletal muscle
STAT3 [100]	miR-9	up-regulation			ESCs
	miR-92a	up-regulation	integrin α5 [133]	down-regulation	muscle, Ecs

* Identified in pig

# Potential target

### Identification of differentially expressed miRNAs between different developmental stages

**Figure 2:**
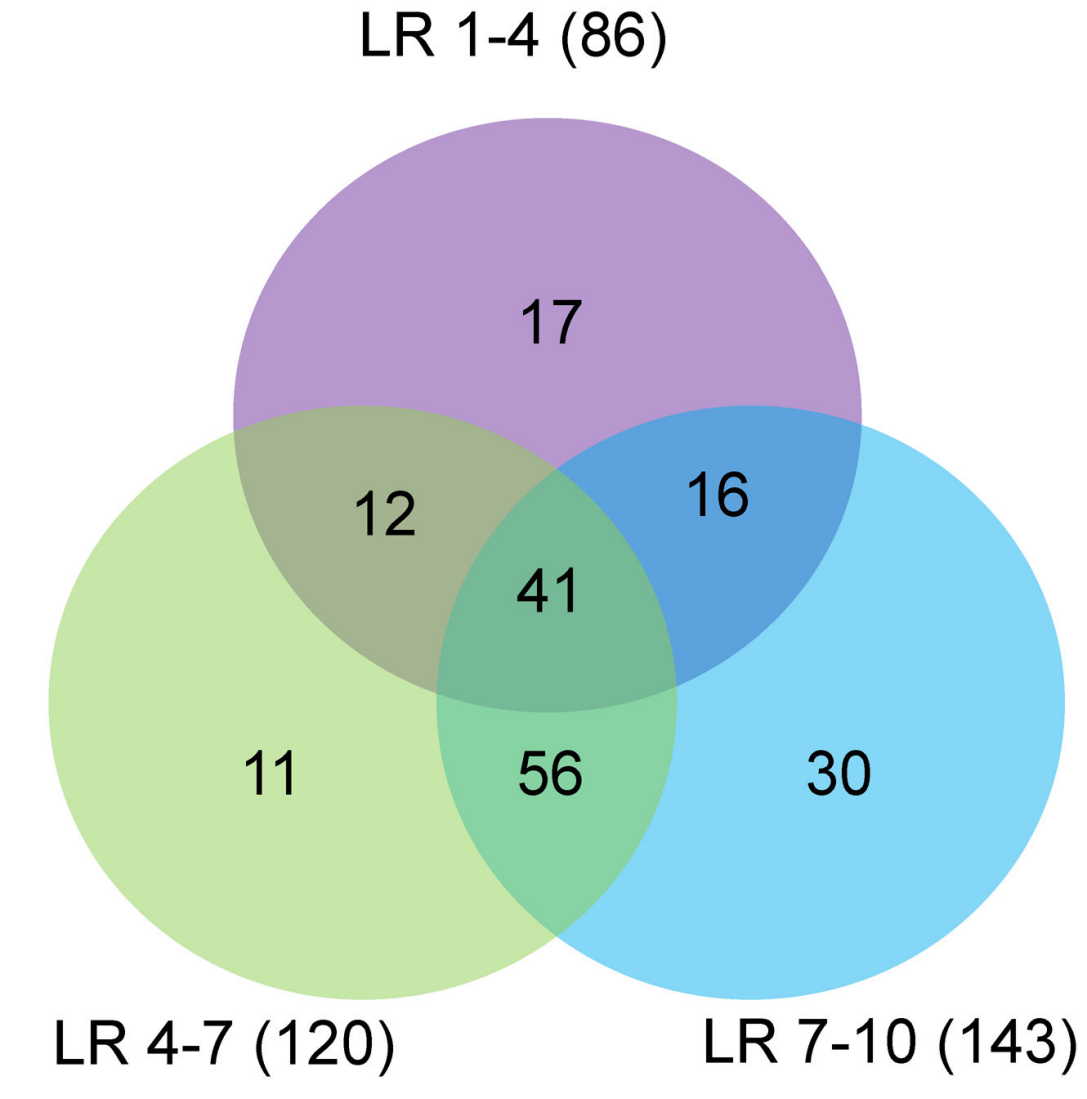
Comparison of differentially expressed (DE) miRNAs among three data sets. The number marked in the overlapping areas shows the common DE miRNAs.

Besides high abundant miRNAs, differentially expressed miRNAs may play important roles in biological processes. To determine miRNAs involved in porcine myogenesis, differentially expressed (DE) miRNAs between two libraries were identified by comparing the normalized expression data of the 257 mature miRNAs. In total, 183 DE miRNAs (│fold-change (log_2_) │≥1; P-value < 0.01) were identified during muscle development (Table 3). Based on previous studies on porcine myogenesis, we speculated that muscle ﬁber formed at 35 to 77 dpc (LR1-4), underwent myofiber transformation at 77 dpc to 28 dpn (LR4-7) and further matured at 28 to 180 dpn (LR7-10) [2–4,6], from which DE miRNAs were obtained and compared. Figure 2 showed that the number of DE miRNAs during myofiber formation was the least but still accounted for almost 50% of total DE miRNAs, from which all of porcine myomiRs (ssc-miR-1, -206, -133 a-3p/a-5p/b) were identified. Notably, there were 53, 57 and 97 DE miRNAs identified from every two data sets, among which 41 DE miRNAs (22% of 183 DE miRNAs) were common, including the above mentioned ssc-miR-133 family.

**Table 3:** The numbers of differentially expressed miRNAs between libraries.

	**Numbers of differentially expressed miRNAs**		
**The comparison between adjacent libraries**	**Total DE miRNAs**	**Total DE miRNAs-up**	**Total DE miRNAs-down**
LR2/1	75	38	37
LR3/2	9	2	7
LR4/3	15	4	11
LR5/4	20	15	5
LR6/5	61	7	54
LR7/6	74	5	69
LR8/7	47	8	39
LR9/8	118	116	2
LR10/9	105	1	104

LR, Landrace; DE, differentially expressed(P<0.01│log2fold-change│>2); up, up regulation; down, down regulation

The variations in abundance might aid understanding of functional miRNAs being involved in myogenesis [21,26]. In addition to the best-studied myomiRs (miR-1, -206 and miR-133 families), 11 other DE muscle-related miRNAs (miR-378 [24], miR-148a [27], miR-26a [28,29], miR-27a/b [30,31], miR-23a [32,33], miR-125b [34], miR-24 [35], miR-128 [36], miR-199a [37] and miR-424 [38]) with high abundance (average RPM >1,000) and another 14 (miR-181a/b/c/d-5p [26], miR-499-5p [11], miR-503 [38], miR-486 [39], miR-214 [40], miR-29a/b/c [41–43], miR-221/222 [44] and miR-208 [11] with low abundance (average RPM <1,000) were detected in myogenesis of pig.

### MicroRNA target predictions and KEGG Orthology analysis

**Figure 3:**
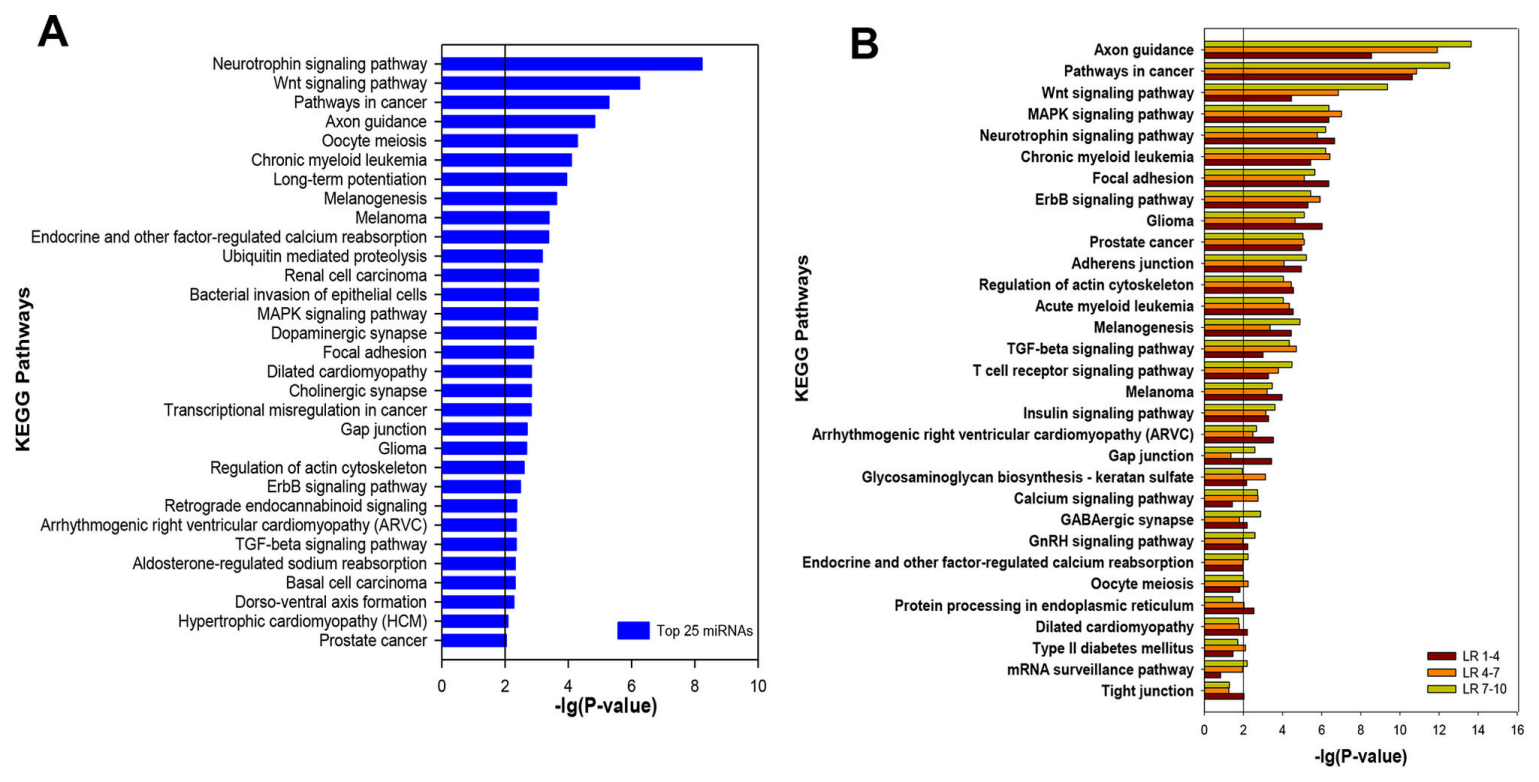
KEGG Orthology analysis of the most abundant miRNAs. **A**. 31 significantly enriched KEGG pathways were achieved using the target genes of the most abundant 25 miRNAs (not including let-7 family) in ten libraries; **B**. comparison of KEGG pathways significantly enriched during skeletal muscle development at 35 to 77dpc (LR 1-4), 77dpc to 28 dpn (LR 4-7) and 28 to 180 dpn (LR 7-10). P-value < 0.01.

To gain further insight into the biological functions of the identified miRNAs, miRecords [17] was applied to predict target mRNAs and KOBAS [18] was used for KEGG Orthology analysis. To identify whether highly expressed miRNAs play key roles in skeletal muscle development, the target genes of 25 miRNAs with the most abundance (not including let-7 family for their ubiquitous expression) in ten libraries (Table 1) were predicted and total 2325 target genes were found. The predictions were then applied to analyze the KEGG Orthology using the KEGG database. Thirty one significantly enriched pathways (P-value < 0.01) were picked out (Figure 3A). Particularly, the highly-expressed miRNAs involved in Wnt [45], MAPK [46], ErbB [47], TGF-beta [48] signaling pathways and Focal adhesion [49], which are known to be closely involved in the regulation of myogenesis. Furthermore, some pathways concerned with diseases such as Melanoma, cancers and Hypertrophic cardiomyopathy (HCM) were actively regulated by miRNAs in muscle tissues [50].

To identify whether different biological processes responding to different stages of muscle development, KEGG Orthology analyses were performed using predicted targets of DE miRNAs at 35 to 77 dpc (LR 1-4), 77 dpc to 28 dpn (LR4-7) and 28 to 180 dpn (LR7-10). Figure 3B illustrated that most pathways with reported function in regulating myogenesis were significantly enriched (P<0.01) in the above three developmental stages, including Wnt, MAPK, Neurotrophin, TGF-beta, ErbB, Insulin, Calcium, GnRH, T cell receptor signaling pathways and Focal adhesion as well as Adherens junction. In particular, two well-known signaling pathways that Wnt and MAPK were both highly enriched in all of the three stages. Comparing these pathways among the three stages, Wnt, the most differently expressed signaling pathway, was significantly enriched throughout the muscle development, with the highest expression at 28 to 180 dpn, the second at 77dpc to 28 dpn, and the least at 35 to 77 dpc. However, MAPK signaling pathway expressed at the same level in all stages. Other pathways such as TGF-beta and Calcium signaling pathways showed more differences in different stages, and Insulin, GnRH and ErbB signaling pathways were just the opposite. Notably, almost all pathways showed s similar expression pattern in two waves of myofiber maturation: myofiber transformation and hypertrophy, suggesting their closer relationship in terms of myogenic regulation.

Furthermore, we refined a more detailed KEGG Orthology analysis using predicted targets of DE miRNAs in Table 3 between two time points contributing to describing the different developmental phases of myogenesis. The significant pathways (P<0.05) related to muscle development were listed in Table 4, most of which have been identified according to the most abundant miRNAs (Figure 3A). Further analysis of these pathways suggested three clusters of enrichment by using SPSS, setting values for statistic significance (^*^P<0.05 and ^**^P<0.01 were assigned 1 and 1.5, respectively, using 0 where no significance was detected) : A) with universal enrichment, including Adherens junction, Focal adhesion, Adipocytokine, GnRH, Insulin, Notch, p53, Phosphatidylinositol, TGF-beta, VEGF and Wnt signaling pathways; B) with more enrichment during prenatal myogenesis, including Axon guidance, Gap junction, Melanogenesis and Neurotrophin signaling pathways; C) with more enrichment during postnatal myogenesis, including Calcium, Chemokine, ErbB, Hedgehog, mTOR, T cell receptor and MAPK signaling pathways (Table 4).

**Table 4:** KEGG Orthology enriched for target genes of DE miRNAs between different libraries.

**Pathway**	**LR2/1**		**LR3/2**		**LR4/3**		**LR5/4**		**LR6/5**		**LR7/6**		**LR8/7**		**LR9/8**		**LR10/9**	
	**up**	**down**	**up**	**down**	**up**	**down**	**up**	**down**	**up**	**down**	**up**	**down**	**up**	**down**	**up**	**down**	**up**	**down**
Adherens junction	*	**		*			*	*		*		**		*	**			**
Adipocytokine signaling pathway				*	*				*	*				**	*			
Axon guidance	*	**	*	**	*	**	*	**	*	**		**	**	**	**			**
Calcium signaling pathway		*	**							*		**		*	**			**
Chemokine signaling pathway												*			*			*
ErbB signaling pathway		**					**	*		**		**	*	**	**			**
Focal adhesion	**	**		*	**	*		**	**	**		**	**	**	**			**
Gap junction		**		**		**	*	**		**		*		**	**			*
GnRH signaling pathway		**	**	*	*	*	**		*	*		**		**	**			**
Hedgehog signaling pathway		*										*		*	*			*
Insulin signaling pathway		**		**		**	**			**		**		**	**			**
MAPK signaling pathway	**	**		*						**		**		**	**			**
Melanogenesis	**	**	**	**	*	**	**	**		**	**	**	*	**	**			**
mTOR signaling pathway		*								**		*			*			*
Neurotrophin signaling pathway	**	**	**		*	**	**	*		**		**	**	**	**			**
Notch signaling pathway							**											
p53 signaling pathway	*				*					*				*	*			*
Phosphatidylinositol signaling system			*			*				**			**		*			*
T cell receptor signaling pathway		**								*		**			**			**
TGF-beta signaling pathway	**	*		**	*			*		**		**	**	**	**			**
VEGF signaling pathway										*								
Wnt signaling pathway	**	**		**		*	**	*		**	*	**	**	**	**			**

Asterisk indicated the significant enrichment of KEGG pathways (* p valued <0.05; **p value < 0.01)

### Clustering miRNA expression

**Figure 4:**
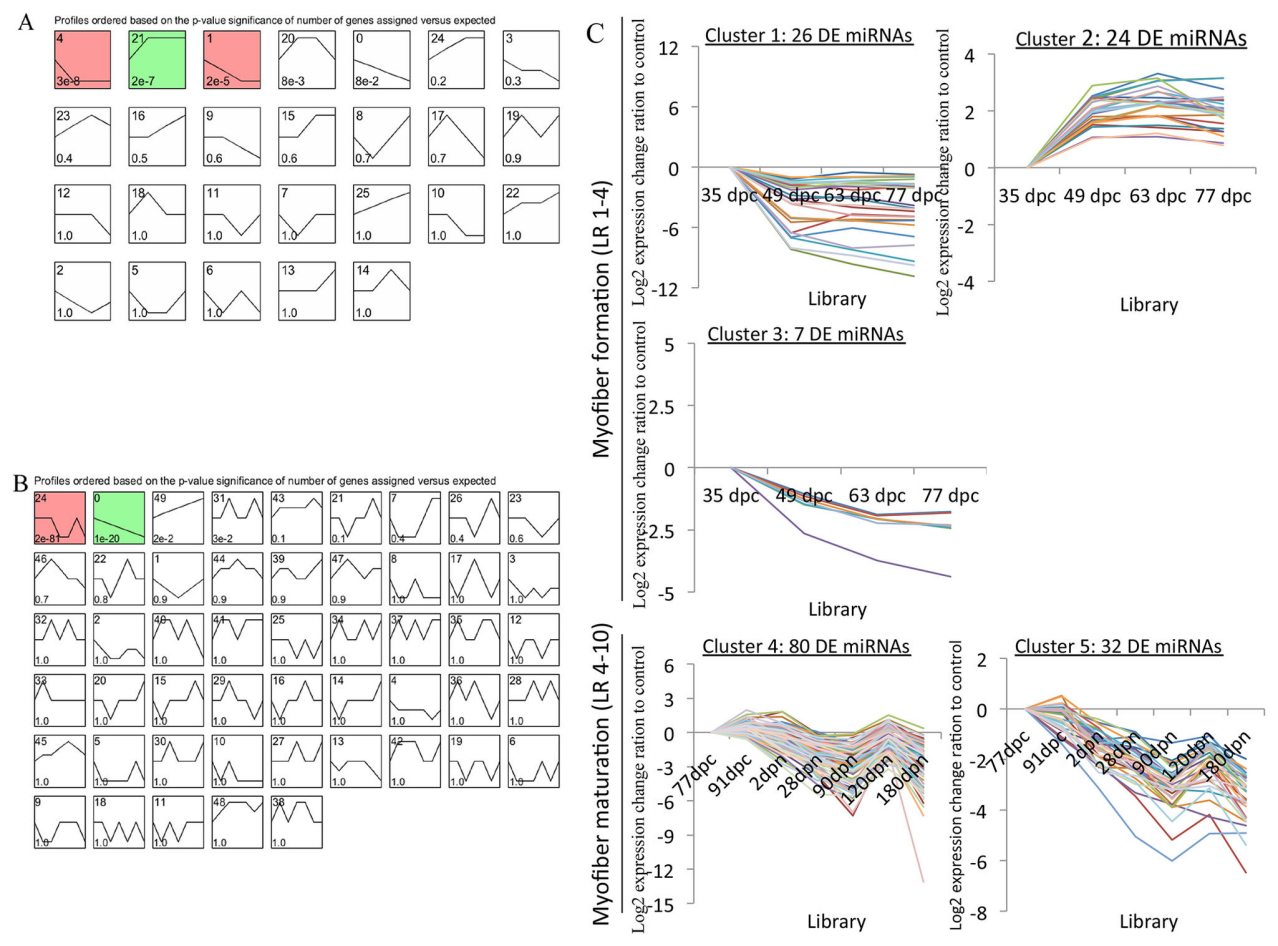
Clustering of miRNA expression profiles. **A**–**B**. Each box corresponded to a model expression proﬁle and only colored proﬁles reached statistical signiﬁcance. The upper-left number in the box gave information about the order of proﬁle (upper left) and the p-value signiﬁcance (bottom left). **A**. 26 clusters of 87 DE miRNAs during myofiber formation, the first three of which showed statistically signiﬁcant; **B**. 50 clusters of 166 DE miRNAs during myofiber maturation, the first two of which showed statistically signiﬁcant. **C**. Five significant clusters of miRNA profiles during myofiber formation (cluster 1-3) and myofiber maturation (cluster 4-5) were displayed as time course plots of log _2_miRNA expression ratios to controls.

We used the Short Time-series Expression Miner (v 1.1, STEM) [51] to cluster temporal profiles of miRNA expression during muscle fiber formation at 35 to 77 doc (LR1-4) as well as fiber further maturation at 77 dpc to 180 dpn (LR4-10). After that a total of 57 out of 87 DE miRNAs (65.5% of 87 DE miRNAs; Table S5) observed at 35 to 77 dpc were significantly clustered into three kinds of expression patterns (11.5% of 26 clusters; Figure 4A), while 112 out of 166 DE miRNAs (67.5% of 166 DE miRNAs; Table S5) observed at 77 dpc to 180 dpn were significantly clustered into two kinds of expression patterns (4% of 50 clusters; Figure 4B, the default P-value was 1E-5). Figure 4C showed miRNA expression profiles for the five clusters found to be significant out of 76 possible clusters successively. The cardinality of each cluster was ranged from 7 DE miRNAs in cluster 3 to 80 in cluster 4 (Table S6). Visual examination of these clusters suggested that down-regulated DE miRNAs were distributed both Cluster 1 and 3 while Cluster 2 included DE miRNAs that showed more gradual increase, which peaked at 63 to 77 dpc during myofiber formation. STEM also clustered biphasic responding DE miRNAs in Cluster 4 and down-regulated DE miRNAs in Cluster 5 during myofiber further maturation from 77 dpc to 180 dpn.

**Figure 5:**
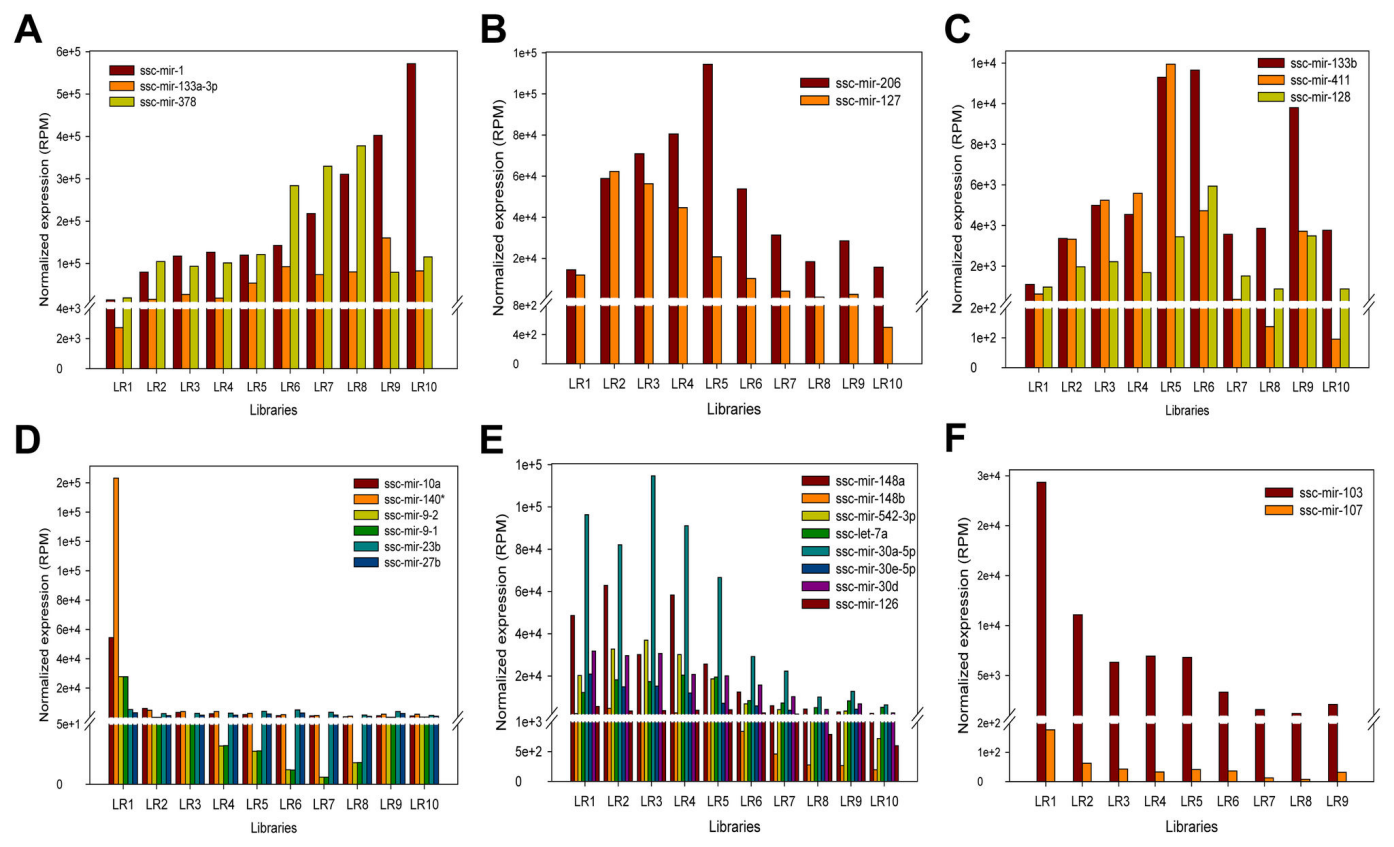
Expression profiles of candidate myogenic miRNAs. Based on STEM results, DE miRNAs that had similar expression profiles with muscle-related miRNAs were selected and clustered into six distinctive types of expression patterns during muscle development.

Similar expression pattern could be associated with functional correlation to a certain extent. Notably, in addition to porcine myomiRs (miR-1, -206, -133 a-3p/a-5p/b), three other miRNAs (miR-128, -208b and -378) reported to be related to muscle development in other mammals were also included in up-regulation Cluster 2, suggesting their roles in the regulation of porcine embryonic myogenesis at 35 to 77 dpc. However, Cluster 5 illustrated that ssc-miR-206 and other four muscle-related miRNAs (ssc-miR-126, -148a/b and -15b) continued to decline while ssc-miR-133b and eleven other muscle-related miRNAs (ssc-miR-125b, -128,-181a/b, -199a, -214, -23a, -24, -424, -503 and -7) in Cluster 4 presented a down and then up trend from 77 dpc to 180 dpn, suggesting their different roles played in adult fiber maturation. Moreover, parts of these miRNA expression profiles during 10 developmental stages were illustrated in Figure 5, providing the possibility for further research into miRNAs that had similar expression profiles with muscle-related miRNAs.

### Validation of the Sequencing data

**Figure 6:**
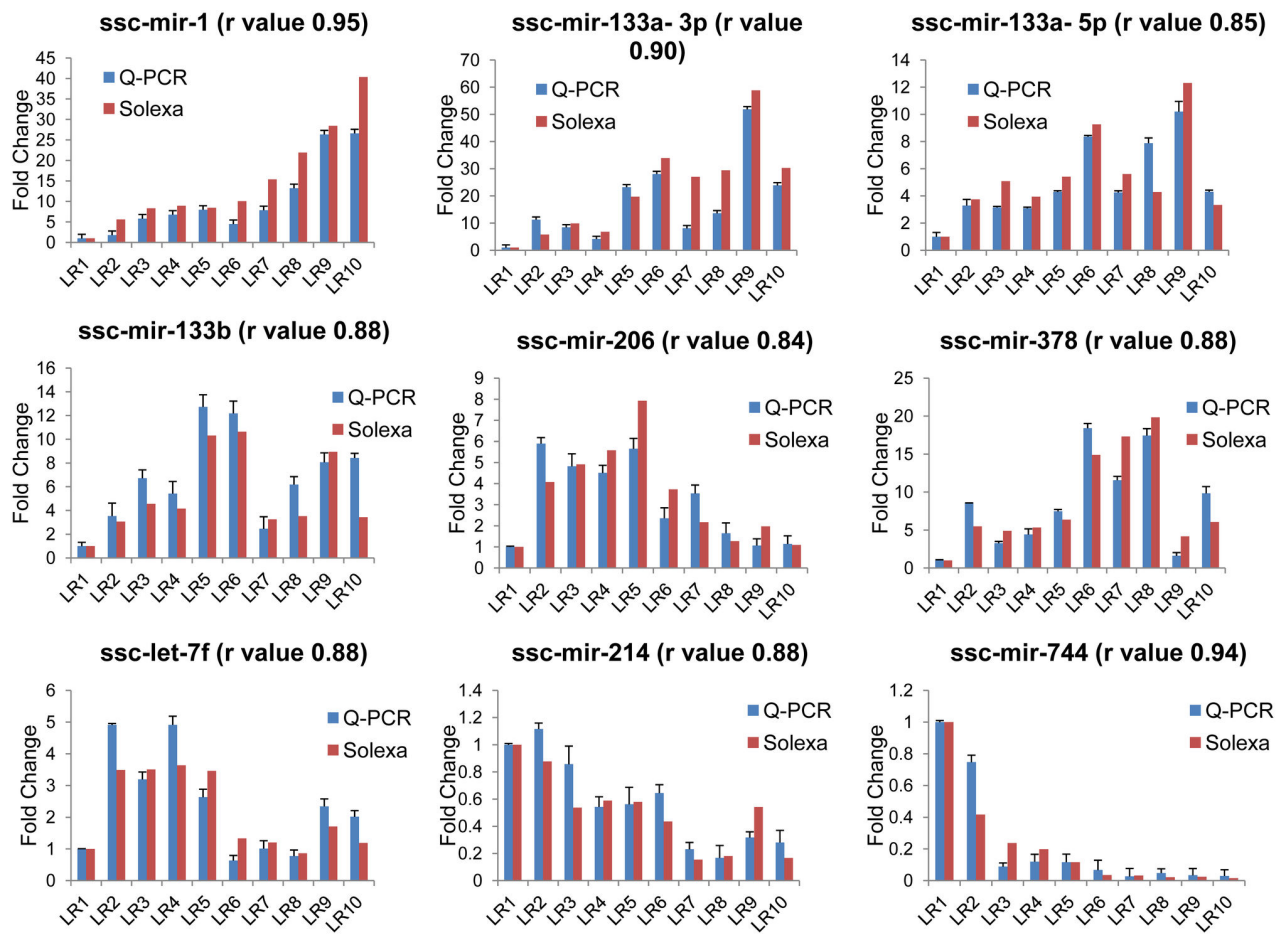
Validation of the sequencing data using Real-time PCR method. The fold changes in abundance of night random miRNAs in 10 libraries were normalized by comparing with the LR1.The *r* value indicates the Pearson Correlation Coefficient between the two methods.

Stem-loop quantitative RT-PCR was applied to validate the sequencing data. Choosing an appropriate set of endogenous control (EC) miRNA genes is crucial, for EC genes are widely used to normalize the miRNA q-PCR data, which are expected to express constantly at all stages of development of one or more tissues. Although U6 snRNA is one of the most widely used internal control, Gu et al. demonstrated that the U6 gene was the least stable gene compared with other candidate EC genes in all tissues (including muscle tissues) comparing with miR–17, -103, -107 and -23a [52]. Hence, the optimal EC gene from above candidates was studied by calculating the standard deviation (*Stdev.*) of four genes in all samples when the fifth was supposed to be an EC gene (Figure S3). As a consequence, miR-17-5p was actually the most stable gene (*Stdev.* = 0.1), while the U6 gene was the least stable gene (*Stdev.* = 4.9), consistent with the previous study [52]. Stem-loop quantitative RT-PCR was then performed on 9 random miRNAs with different expression levels (miR-1, -206, -133a-3p, -133a-5p, -133b, -378, -214, -744 and let-7f) to validate the sequencing data (Figure 6). Stem-loop RT-PCR primers were presented in Table S7. The Pearson correlation coefficient of the Real-time PCR and the Solexa sequencing was calculated and the *r* values ranged from 0.84 to 0.95, indicating that there was a high consistency between the two methods.

## Discussion

Since pigs are important farm animals and are suitable models for studying human diseases, we herein presented the systematical porcine miRNAome at ten important developmental stages of skeletal muscle using Solexa sequencing. A total of 247 known and 297 potential novel miRNAs were identified. 247 known porcine miRNAs were substituted as 257 mature porcine miRNAs in miRBase 18.0 for further analysis. Similar to the work of McDaneld et al. [14], this study aimed at studying the mechanism of skeletal muscle development in swine through analysis of miRNA transcriptome profiles at designed different developmental stages. In addition, we both focused on the changes in abundance of myomiRs (miR-1, -206 and -133) during swine skeletal muscle development. Contrarily, different developmental stages were used to study muscle development in the two investigations. Moreover, the proliferating satellite cells were employed in the previous work, which might be complement each other and contribute to revealing the expression profiles and biological function of miRNA during swine skeletal muscle development more comprehensively. Unlike the previous work, the present study paid more attention to the most abundant miRNAs, but not limited to the well-studied myomiRs, which have been proven to play important roles during swine skeletal muscle development, consistent with the conclusion that functional miRNAs tend to be highly expressed [19]. The sequencing data was then verified by stem–loop quantitative RT-PCR. Moreover, the abundance of the top 15 miRNAs were found to be the majority of total reads in each library (Figure S2). KEGG Orthology analysis of the most abundant 25 common miRNAs (excluding let-7 family) from 10 libraries indicated that their involvement in the regulation of canonical myogenesis-related pathways and diseases (Figure 5). Intriguingly, 11 of the 25 common miRNAs were reported to function as muscle-related miRNAs including the well-known myomiRs (miR-1, -206 and -133 family). Therefore, it is reasonable to hypothesize that the functional miRNA, in terms of mediating target suppression to regulate myogenesis and muscle diseases, tended to express abundantly.

Currently, differently expressed (DE) miRNAs were thought to be closely related to almost all aspects of muscle development. In this study, a total of 183 DE miRNAs between different libraries were identified. Ssc-miR-1 was the most abundant miRNA in ten libraries, consistent with the well-established function of miR-1 during skeletal muscle development [21]. Interestingly, the second abundant DE miRNA was miR-378, a new candidate miRNA for myogenesis in pigs by down-regulating porcine BMP2 or MAPK1 [53]. Another DE miRNA (miR-148a), whose average abundance before birth was eight times higher than that in postnatal, might be a part of mechanism implicated in differences between embryonic myogenesis and adult myofiber maturation. Exceptions were some previously reported muscle-related miRNAs such as miR-181c/d-5p, miR-29b/c, miR-221/222 and miR-208, all of which expressed at a very low level (average RPM <100). In addition to highly expressed myomiRs, the expression of muscle-related miRNAs was varied in pigs, suggesting that highly expressed muscle-related miRNAs that have been validated in other species should be the first priority in studying porcine skeletal muscle development, followed by the muscle-related miRNAs expressed at lower levels.

Next, target prediction and KEGG Orthology analysis of stage-specific DE miRNAs might suggest that myofiber formation and maturation were controlled by multiple signal pathways in different ways, suggesting that different molecular regulation mechanism underlined different biological processes. In particular, the two waves of myofiber maturation: myofiber transformation and hypertrophy showed a closer relationship in myogenic regulation, providing a possibility to undergo subsequent STEM clustering by combining them. Furthermore, a more detailed KEGG Orthology analysis of DE miRNAs between two time points identified a list of pathways participating in the regulation of myogenesis and related diseases, suggesting that DE miRNAs were tightly related with biological processes at different stages of muscle development. For example, Wnt signaling pathway involved in embryonic myogenesis and in regulating the homeostasis of adult muscle [45], resulting its universal enrichment from embryonic to adult myofiber maturation. Calcium signaling, though with less attention, was considered potential mediators of postnatal muscle development and hypertrophy [54], giving some indication for more enrichment during postnatal myogenesis. An exception was Notch signaling pathway, though it was classified to the category with universal enrichment, only expressed highly during late embryogenesis. However, previous studies have showed its crucial role not only for embryogenesis but for muscle maintenance and repair in the adult [55]. One reason might explain the difference is that there is still plenty of unknown information about miRNA, resulting insufficiency of its functional annotation. In general, integrated analysis of sequencing results and biological pathway information might add insight into the vital roles played by miRNA during skeletal muscle development. However, comprehensive information about miRNA is still lacking, such as insufficiency of functional analysis, which requires more research focusing on combination of bioinformatic analysis of miRNAome and subsequently experimental validation.

We hypothesized that miRNAs with similar expression patterns might be function-related. STEM clustering results suggested that ssc-miR-378 functioned as a new candidate miRNA for porcine myogenesis because of its expression profile similar to ssc-miR-1 and -133a-3p (Figure 5A). Interestingly, recently studies have validated that ssc-miR-378 regulated myogenesis by directly targeting the BMP2 or MAPK1 in pig, suggesting that the STEM clustering was reliable for analyzing miRNA expression profiles as well as for predicting of candidate myogenic miRNAs. We focused on either highly expressed DE miRNAs or muscle-related miRNAs to ensure the accuracy of prediction. Consequently, 18 candidate miRNAs were selected, including ssc-miR-378, -127, -128, -411, 23b, -27b, -10a, -140*, -9-1/-2, -148a/b, -126, 542-3p, 30a-5p/d/e-5p and miR-103 (Figure 5). MiR-127, which is located within a CpG island with little research on myogenesis, showed similar expression pattern to ssc-miR-206, up regulated at 35 to 77 dpc and down regulated at 77 dpc to 180 dpn (Figure 5B), providing the possibility for further investigation concerning the function of ssc-miR-127 during muscle development. miR-128 was reported to participate in the regulation of adipogenesis, osteogenesis and myogenesis [36] and herein, together with ssc-miR-411, up regulated at 35 to 77 dpc and fluctuated at 77 dpc to 180 dpn (Figure 5C), might behave in a similar manner as ssc-miR-133b during porcine muscle development. MiR-23b was found to interact with TGF bata signaling by down-regulating Smads in fetal hepatocytes [56], while miR-27b was involved in myogenic differentiation [31] as well as fast-specific and glucocorticoid-dependent myostatin expression [30], both of which were highly expressed at 35 dpc and down-regulated thereafter (Figure 5D), suggesting their roles played in porcine embryonic myogenesis. Like ssc-miR-23b and 27b, it is reasonable to hypothesize that ssc-miR-10a, -140* and -9-1/-2 might act on earlier embryonic myogenesis, for they highly expressed at 35 dpc then decline dramatically. MiR-148a has been identified as a novel myogenic miRNA that mediated myogenic differentiation via targeting ROCK1 [27], while miR-126 attenuated insulin signaling [57] and governed vascular integrity and angiogenesis [58], suggesting their interactions with signaling pathways were required for muscle normal development and maintenance. Similarly, ssc-miR-148b, -542-3p and -30 family (a-5p/d/e-5p) showed similar expression patterns with ssc-miR-148a and -126, highly expressed and down-regulated at 77 dpc to 180 dpn (Figure 5E), making it possible that they belong to the candidate myogenic miRNAs. MiR-103/107 family was validated to attenuate miRNA biosynthesis by targeting Dicer [59], regulate insulin sensitivity through down-regulating caveolin-1 [60] and affect cellular migration by modulating CDK5R1 expression [61], allowing us to hypothesize that these miRNA-mediated mechanism may affect myogenesis when the microRNA family, especially ssc-miR-103, down-regulated during both myofiber formation (at 35 to 77 dpc) and maturation (at 77 dpc to 180 dpn) (Figure 5F). In summary, STEM clustering of miRNA temporal expression contributed to determining candidate myogenic miRNAs in an effective way.

In this study, 297 novel miRNAs were identified. Analysis of the evolutionary conservation of these novel miRNAs revealed that none is conserved in mammals. Therefore, it is reasonable to hypothesize that pig-specific miRNAs may exist. However, it is the first time that more than 200 novel miRNAs were detected from miRNAome of porcine skeletal muscle attributing to high-throughput deep sequencing technology. Although few of these novel miRNAs might play any roles in establishing and maintaining phenotype of muscle tissue during individual development for their extremely low expression, there are still several highly expressed novel miRNAs that need further validation. In particular, a functional validation of novel and species-specific miRNAs is a challenge for understanding the critical roles played by miRNAs during muscle development and for providing the valuable information for pig meat quality improvement.

## Supporting Information

Figure S1 **Saturation plots of ten libraries.**
(TIF)

Figure S2 **Counts characteristics of the unique miRNAs in each library.** Starting from the miRNA with the highest counts (x-axis), the black bar represents the accumulative proportion of miRNAs in total counts of each library. The red horizontal line represents the proportion of individual miRNA versus the total 257 miRNAs.
(TIF)

Figure S3 **Determination of the optimal endogenous control (EC) pig genes for normalization.** Based on standard deviation, the stability of 5 candidate EC genes was measured. The pairwise difference between all tested genes was compared using *t-test*, and the significance was labeled with asterisk (^**^p value < 0.001).
(TIF)

Table S1 **Annotations of sequenced small RNAs.** Table S1-1 Number of small RNA reads. Table S1-2 Summary of reads matching noncoding RNA. Table S1-3 Summary of small RNA matching noncoding RNA databases.
(XLS)

Table S2 **Summary of predicted novel miRNAs.** Table S2-1 Predicted chromosomal positions and counts of novel miRNAs. Table S2-1 Predicted chromosomal positions and counts of novel miRNAs.
(XLS)

Table S3 **Summary of reads distribution of 257 known porcine miRNAs.** Table S3-1 Expression profiles of 257 miRNAs in ten libraries. Table S3-2 Raw reads distribution. Table S3-3 The distribution of numbers for normalized miRNAs.
(XLS)

Table S4 **Summary of top 15 most abundant miRNAs in 10 libraries.**
(XLS)

Table S5 **Expression profiles of DE miRNAs during LR 1-4 and LR 4-10.**
(XLS)

Table S6 **Information about five significant clusters.**
(XLS)

Table S7 **Primers for miRNA RT-qPCR.**
(XLS)
