# Supplementary figures and images for: Integrative Analysis of Porcine microRNAome during Skeletal Muscle Development
Source: PLoS One. 2013 Sep 11;8(9):e72418. doi: 10.1371/journal.pone.0072418 (PMC3770649; doi:10.1371/journal.pone.0072418)

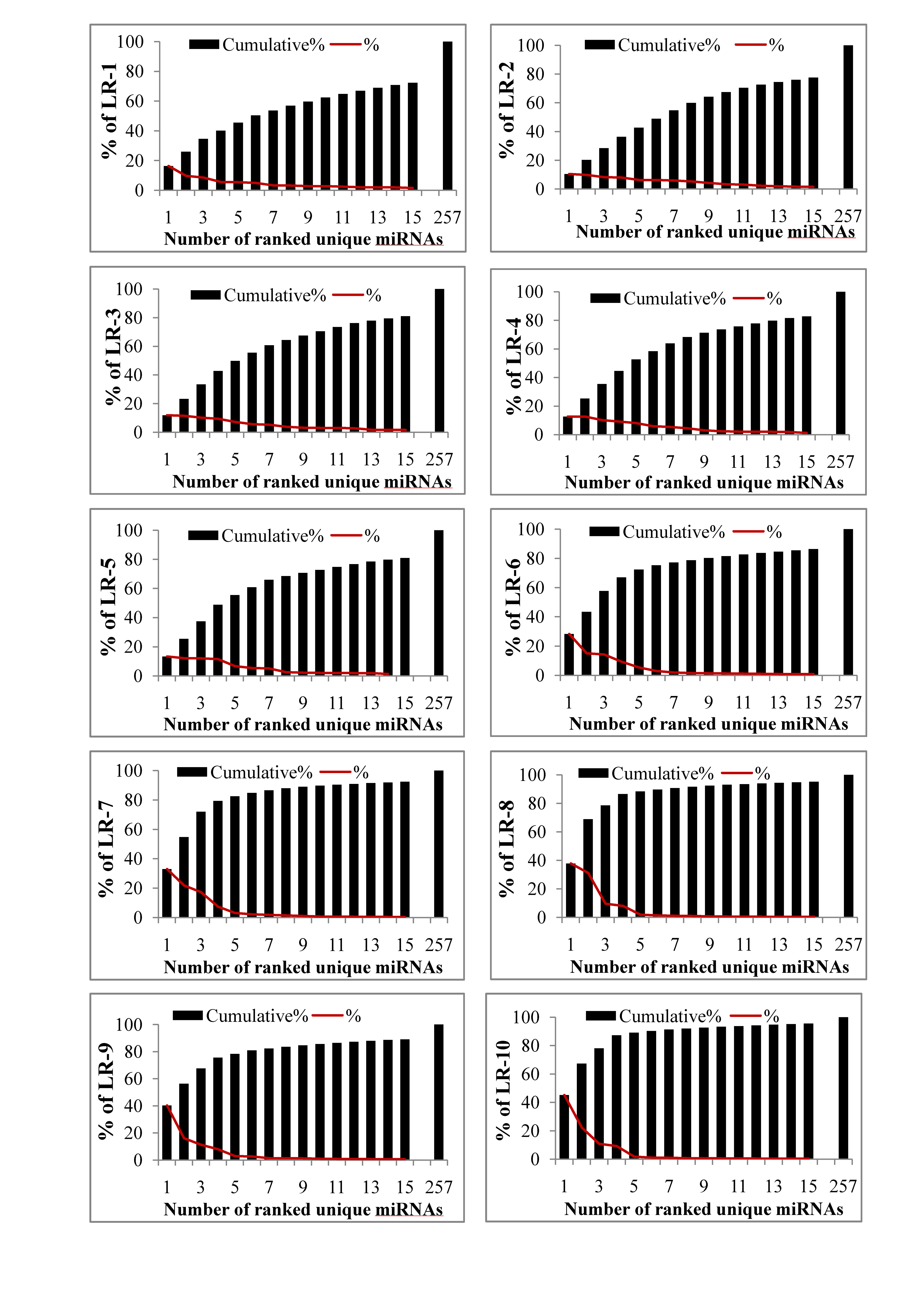

Supplement: Figure S2 — Counts characteristics of the unique miRNAs in each library. Starting from the miRNA with the highest counts (x-axis), the black bar represents the accumulative proportion of miRNAs in total counts of each library. The red horizontal line represents the proportion of individual miRNA versus the total 257 miRNAs. (TIF) [file pone.0072418.s002.tif]

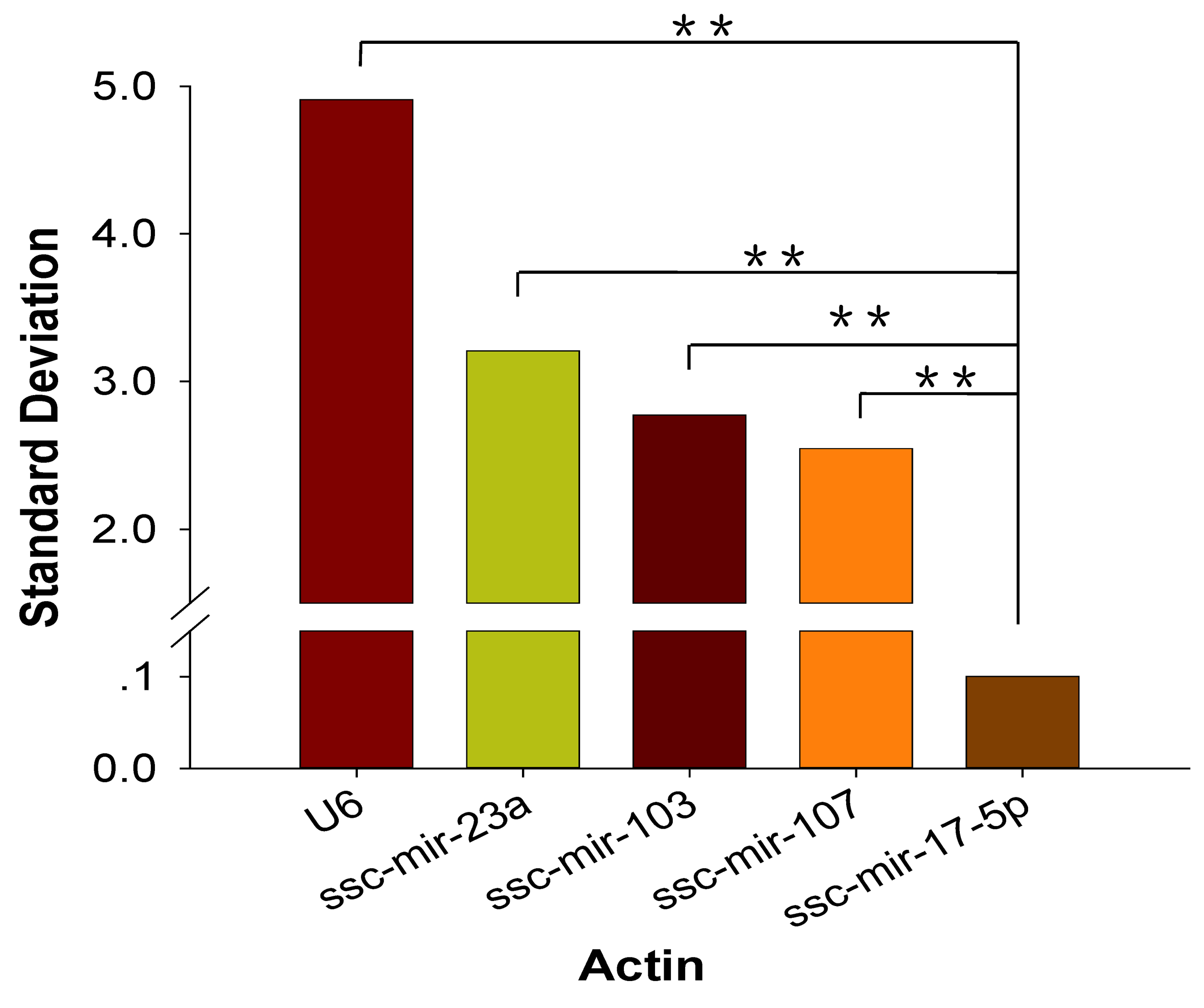

Supplement: Figure S3 — Determination of the optimal endogenous control (EC) pig genes for normalization. Based on standard deviation, the stability of 5 candidate EC genes was measured. The pairwise difference between all tested genes was compared using t-test, and the significance was labeled with asterisk (**p value < 0.001). (TIF) [file pone.0072418.s003.tif]
